# A method for generating highly multiplexed ChIP-seq libraries

**DOI:** 10.1186/1756-0500-7-312

**Published:** 2014-05-22

**Authors:** Ethan Ford, Chrysa Nikopoulou, Antonis Kokkalis, Dimitris Thanos

**Affiliations:** 1 Biomedical Research Foundation, Academy of Athens, 4 Soranou Efesiou Street, Athens 11527, Greece

**Keywords:** Chromatin immunoprecipitation, ChIP-seq, Next generation sequencing, Genomics, Epigenetics

## Abstract

**Background:**

The barcoding of next generation sequencing libraries has become an essential part of the experimental design. Barcoding not only allows the sequencing of more than one sample per lane, but also reduces technical bias. However, current barcoding strategies impose significant limitations and/or technical barriers in their implementation for ChIP-sequencing.

**Findings:**

Converting Y-shaped sequencing adapters to double stranded DNA prior to agarose gel size selection reduces adapter dimer contamination and quantitating the number of cycles required for amplification of the library with qPCR prior to library amplification eliminates library over-amplification.

**Conclusions:**

We describe an efficient and cost effective method for making barcoded ChIP-seq libraries for sequencing on the Illumina platform.

## Findings

Chromatin immunoprecipitation followed by next-generation sequencing (ChIP-seq) is a powerful method to identify the genome-wide binding profiles of chromatin-associated proteins and has emerged as one of the most important tools used to study transcriptional regulation, DNA replication, DNA recombination and chromatin structure [1].

Technological advancements in next-generation sequencing throughput have facilitated the production of more sequencing data per lane on the Illumina platform than what is necessary to achieve saturation in a ChIP-seq experiment. Thus, in order to perform experiments in cost effective manner, multiple barcoded ChIP-seq libraries must be pooled together and sequenced in a single lane. In addition, pooling barcoded samples from the same or different experiments reduces technical variability between samples [2]. Thus, barcoding not only reduces costs, it also produces higher confidence and quality data. Unfortunately, current barcoding strategies impose significant limitations and/or technical barriers to their implementation for ChIP-seq analysis.

There are currently two main barcoding strategies for the generation of NGS (next generation sequencing) libraries for sequencing on the Illumina platform. The first embeds the barcode in the adapter oligonucleotide used for library construction so that the first nucleotides sequenced corresponds to the barcode sequence [3]. On the Illumina platform, it is critical that the four bases (A, T, G and C) are represented in roughly equal proportions in the first nucleotides sequenced [4]. Thus, it is necessary to pool libraries in multiples of four so that the nucleotide composition is balanced. However, this causes inflexibility in that only multiples of four samples must be used, and also limits the researcher from pooling libraries in unequal ratios if more reads are required from one sample over another. In addition, the invariant thymidine residue at the 3’ end of the oligonucleotide adapters, which is required for ligation to the immunoprecipitated DNA, can have an adverse effect on the quality of the sequences generated. To address these issues it is usually recommended that libraries are spiked with Illumina’s PhiX control library and/or sequenced at a lower density [5]. However, these methods reduce sequencing throughput significantly.

Because of the difficulties associated with the incorporation of the barcode at the beginning of the sequencing read, Illumina has implemented a second-read barcoding strategy as a centerpiece of its TruSeq technology. The second read strategy circumvents the problems associated with having the barcode embedded at the beginning of the sequencing read, however current adapter design makes it difficult to implement this strategy using DNA from ChIP-seq experiments. Specifically, during library preparation adapter dimers are formed and must be removed from the library before sequencing. Size selection with AMPure XP beads is an efficient method to remove adapter dimers [5], but since ChIP libraries often contain DNA in the size range of 100 to 200 bp, the size difference between the adapter dimers and adapter-ligated immunoprecipitated DNA is difficult to resolve with this method. Size selection by agarose gel electrophoresis, in principle, can resolve the size difference between adapter-dimers and adapter-ligated immunoprecipitated DNA, but the large Y-shaped DNA adapter molecules required for second read barcoding run aberrantly through agarose gels.

**Figure 1:**
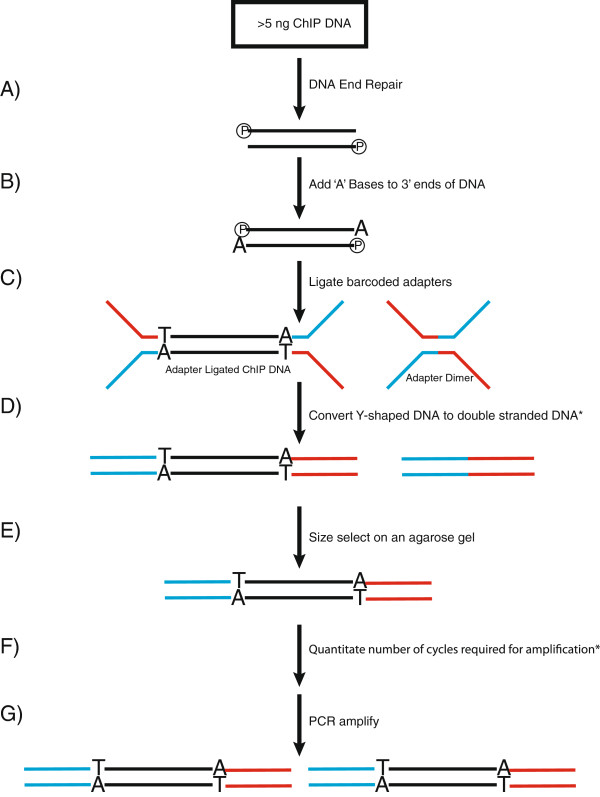
**A schematic representation of the method for making barcoded ChIP**-**seq libraries.** An asterisk denotes novel steps in the protocol. **(A)** End-repair. **(B)** A-tailing. **(C)** Adapter ligation. **(D)** Conversion of Y-shaped DNA to double-stranded DNA. **(E)** Size selection. **(F)** Cycle quantitation. **(G)** PCR amplification.

Here, we describe a method that bypasses the problems associated with size selection. Specifically, compared to the two methods discussed, we perform five cycles of PCR prior to size selection on an agarose gel to convert the Y-shaped DNA to double-stranded DNA, so that both the adapter-dimers and adapter-ligated immunoprecipitated DNA run true to their size during agarose gel electrophoresis and accurate size selection can be performed. This step also results in a modest amplification of the immunoprecipitated DNA prior to agarose gel size selection, which increases the yield of an otherwise inefficient step in the library preparation protocol. In addition, we also include a method to accurately quantitate the number of cycles required for PCR amplification of the library, which reduces biases caused by over-amplification [6]. Library amplification is performed using Kapa HiFi polymerase, which is more efficient and produces less bias than Phusion polymerase [7]. Finally, we have reduced the amount of reagents required for library construction making library construction more affordable, which is especially important as sequencing costs drop.A schematic step-wise representation of the method is illustrated in Figure 1. Briefly, a minimum of 2 ng of immunoprecipitated DNA is treated with T4 DNA polymerase, Klenow fragment and T4 polynucleotide kinase to blunt the DNA ends and add 3’ phosphates (Figure 1A). Next, single 5’ adenine overhangs are generated by incubating the DNA with Klenow exo-minus and dATP (Figure 1B). Barcoded Y-shaped oligonucleotide adapters are ligated to the DNA (Figure 1C). The DNA molecules are then converted to double-stranded DNA with five cycles of PCR (Figure 1D), so that the DNA molecules run predictably during the subsequent size selection by agarose gel electrophoresis (Figure 1E). The precise number of PCR cycles required for amplification is determined by qPCR (Figure 1F) and finally the library is amplified by PCR accordingly (Figure 1G).

**Figure 2:**
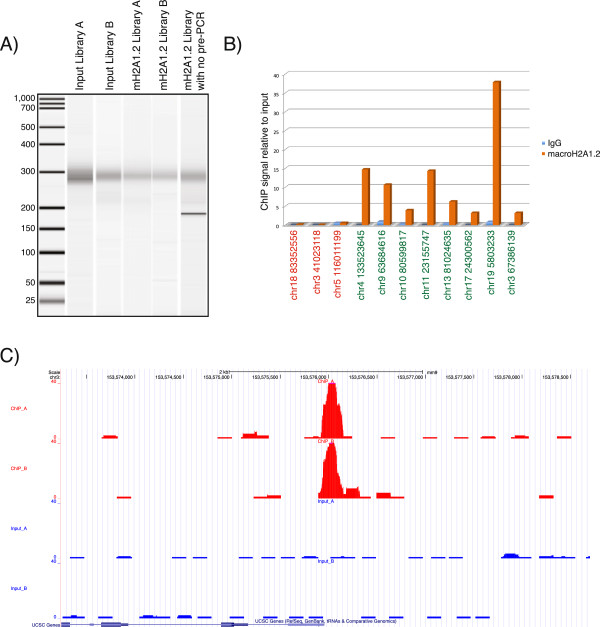
**Validation of the ChIP**-**seq library construction method by ChIP**-**seq against macroH2A1.2.(A)** Bioanalyzer 2100 gel image showing 1 ul of each library run on DNA 1000 chip **(B)** Validation of several macroH2A1.2 peaks by qPCR at the respective loci. Three loci not bound by macroH2A1 are on the left side (red) and eight loci identified as macroH2A1 peaks are on the right side (green). Loci are named according to their location on the mm9 mouse genome build. **(C)** A UCSC genome browser screen shot of representative macroH2A1.2 peaks. Input DNA is colored in blue and the two anti-macroH2A1.2 ChIP libraries A and B in red.

To demonstrate the robustness of our library preparation protocol we performed ChIP against the histone variant macroH2A1 in mouse embryonic fibroblasts (MEFs). MacroH2A1 is a unique histone variant, in that in addition to the canonical histone H2A domain, it also contains a relatively large (30 kD) amino-terminal ‘macro’ domain. While macroH2A1 is generally associated with gene repression, it is also required for the activation of a subset of genes [8]. The alternative use of a 64 bp or 73 bp mutually exclusive exon results in the production of two distinct proteins, macroH2A1.1 and macroH2A1.2, respectively [9]. While macroH2A1.1 efficiently binds the NAD^+^ metabolite ADP-ribose, macroH2A1.2 does not [10]. We prepared Illumina libraries from immunoprecipitated material using antibodies targeted to macroH2A1.2 as well as input DNA (for a detailed protocol see Additional file 1). Five ChIP-seq libraries were made, two from the macroH2A1.2 immunoprecipitated DNA (macroH2A1.2 library A and macroH2A1.2 library B), two from the input DNA (input library A and input library B) and one from input DNA in which the pre-agaros gel PCR step was omitted. The libraries were amplified with 12 cycles of PCR (including the pre-size selection PCR cycles) and visualized on the Agilent Bioanalyzer (Figure 2A) before being sequenced in one lane of the HiSeq2000. In total, 76 million reads were obtained and demultiplexed with Illumina’s Casava data analysis pipeline. 24 million, 15 million, 21 million and 15 million reads for macroH2A1.2 library A, macroH2A1.2 library B, input library A and input library B respectively, were mapped to the mouse genome build mm9 with the Bowtie short read alignment software. MacroH2A1.2 enriched regions were identified with the MACS software package [11] and the data sets were uploaded onto the UCSC genome browser. In total we identified 33 peaks in macroH2A1.2 library A. Every peak identified in macroH2A library A was also a peak in macroH2A library B, demonstrating the reproducibility of the library construction protocol. An example peak is shown in Figure 2C as a UCSC genome browser screenshot. A few of the identified peaks were validated by ChIP-qPCR (Figure 2B) further supporting the specificity and the effectiveness of the method. Adapter sequences represented less than 1% of sequences, which highlights the effectiveness and necessity to convert the Y-shaped adapters to double-stranded DNA prior to agarose gel size selection.

In conclusion, we present a robust and economical method for generating barcoded ChIP-seq libraries, which has substantial advantages over current methodologies. We have successfully pooled between two and eight samples in a single lane of the HiSeq2000 and consider that any number of samples could be pooled without difficulties. The libraries are compatible with Illumina’s TruSeq platform and can be demultiplexed by Illumina’s Casava data analysis pipeline. Importantly, the two innovations detailed in this method, that is the conversion of the Y-shaped adapter DNA to double-stranded DNA prior to agarose gel size selection and the determination of the precise number of cycles to perform during PCR amplification can also be applied to other next-generation sequencing techniques such as MeDIP-seq.

## Abbreviations

ChIP: Chromatin immunoprecipitation; NGS: Next-generation sequencing; PCR: Polymerase chain reaction; MeDIP-seq: Methylated DNA immunoprecipitation.

## Competing interests

The authors declare no competing interests.

## Authors’ contributions

EF developed the method, designed the experiments, analyzed the data and co-wrote the manuscript. CN and AK performed the experiments and co-wrote the paper. DT supervised the project and co-wrote the manuscript. All authors read and approved the final manuscript.

## Acknowledgements

We thank S. Tsiftsoglou, A. Banos and M. Lavigne for critical reading of the manuscript.

### Role of the funding sources

This work was supported by a FP6 mobility Marie Curie International Incoming Fellowship grant to EF, a Cooperation grant from the Greek Secreteriat for Research and Technology (EDGE) and a KMW offsets grant to DT.

## Supplementary Material

Additional file 1 **Detailed protocol.** Chromatin immunoprecipitation and multiplexed illumina library preparation protocol. Click here for file
