# Supplementary material for: A method for generating highly multiplexed ChIP-seq libraries
Source: BMC Res Notes. 2014 May 22;7:312. doi: 10.1186/1756-0500-7-312 (PMC4048252; doi:10.1186/1756-0500-7-312)
Supplement: Additional file 1 — Detailed protocol. Chromatin immunoprecipitation and multiplexed illumina library preparation protocol. [file 1756-0500-7-312-S1.pdf]

# Detailed Protocol

## Chromatin immunoprecipitation and multiplexed Illumina library preparation protocol

**Protocol For:**

A method for generating highly multiplexed ChIP-seq libraries

Ethan Ford, Chrysa Nikopoulou, Antonis Kokkalis and Dimitris Thanos

Biomedical Research Foundation, Academy of Athens, 4 Soranou Efesiou Street, Athens 11527, Greece

## REAGENTS

**AMPure XP beads** (Beckman Coulter, product # A63881)
**Polyethylene glycol average M.W. 8000** (Sigma, product # 89510-250G-F)
**Qubit dsDNA HS assay kit** (Life Technologies, product # Q32851)
**Qiagen minElute PCR purification kit** (Qiagen, product # 28004)
**Qiagen minElute gel extraction kit** (Qiagen, product # 28604)
**T4 DNA polymerase, 3 units/µl** (New England Biolabs, product # M0203S)
**Klenow fragment, 5 units/µl** (New England Biolabs, product # M0210S)
**T4 polynucleotide kinase, 10 units/µl** (New England Biolabs, product # M0201S)
**Klenow 3' to 5' exo minus, 5 units/µl** (New England Biolabs, product # M0212S)
**2X Kapa Biosystems HiFi Library Amplification Kit** (Kapa Biosystems, product # KK2611)
**KAPA SYBER FAST 2x Universal qPCR master mix** (Kapa Biosystems, product # KK4601)
**MetaPhor agarose** (Lonza, product # 50180)
**SeaKem LE agarose** (Lonza, product # 50000)
**ethidium bromide** (Applichem)
**EDTA** (Applichem)
**glycerol** (Applichem)
**Tris base** (Applichem)

## PROCEDURE

### Library Preparation

1. Mix 10 ng ChIP DNA
   5 µl 10x NEB T4 DNA ligase buffer
   2 µl 10 mM dNTPs
   0.5 µl End Repair Enzyme Mix
   $H_2O$ to make final volume 50 µl
2. Incubate at 20˚ C for 30 min in PCR machine.
3. Add 50 µl AMPure XP beads and 50 µl 30% $PEG_{8000}$ 1.25 M NaCl
4. Incubate at room temperature for 15 min.
5. Place on magnetic rack for 5 min.
6. Remove and discard supernatant. When removing supernatant do so very slowly with Pipetman being careful not to take any beads.
7. Keep sample in magnetic rack and add 200 µl of freshly prepared 80% ethanol.
8. Incubate for 30 seconds. Remove and discard all supernatant.
9. Repeat steps 7 and 8 one more time.
10. Let the beads dry at room temperature for 2 min.
11. Add 18 µl TE/10 and pipet up and down 10 times.
12. Incubate at room temperature for 2 min.
13. Place in magnetic rack for 5 min.
14. Transfer 16.5 µl of the supernatant to a new 0.2 ml PCR tube. Again it is important to remove the supernatant very slowly being careful not to

take any beads. If you accidentally remove some beads, pipet sample back into same tube and wait for the beads to separate again.

15. Mix 16.5 μl End-repaired DNA (from step 14)
    2 μl 10X NEB Buffer #2
    1 μl 4 mM dATP
    0.5 μl 5 U/μl Klenow 3' to 5' exo minus (NEB)
16. Incubate at 37˚ C for 30 min.
17. Mix 20 μl 'A'-tailed DNA (from step 16)
    25 μl 2x Quick Ligase Buffer (NEB)
    1 μl Annealed oligonucleotide adapters (0.25 μM)
    1 μl $H_2O$
    1.5 μl 2,000 U/μl T4 DNA ligase (NEB)

    Note: The quantity of adapters stated here is a good amount if you are starting with 10 ng of DNA. If you are starting with more or less DNA it is a good idea to scale the amount of oligonucleotide adapters you are using proportionally.

18. Incubate at room temperature for 20 min.
19. Add 5 μl 0.5 M EDTA, pH 8.0
20. Add 50 μl AMPure XP beads and 50 μl 20% $PEG_{8000}$ 1.25 M NaCl
21. Incubate at room temperature for 15 min.
22. Place on magnetic rack for 5 min.
23. Remove and discard supernatant. When removing supernatant do so very slowly with Pipetman being careful not to take any beads.
24. Keep sample in magnetic rack and add 200 μl of freshly prepared 80% ethanol.
25. Incubate for 30 seconds. Remove and discard all supernatant.
26. Repeat steps 24 and 25 one more time.
27. Let the beads dry at room temperature for 2 min.
28. Add 25.5 μl TE/10 and pipet up and down 10 times.
29. Incubate at room temperature for 2 min.
30. Place in magnetic rack for 5 min.
31. Transfer 24 μl of the supernatant to a new 0.2 ml PCR tube.

    Note: It is important to remove the supernatant very slowly being careful not to take any beads. If you accidentally remove some beads, pipet sample back into same tube and wait for the beads to separate again.

32. On ice mix 24 μl Adapter-ligated DNA (from Step 31)
    1 μl PCR primer mix (25 μM)
    25 μl 2X Kapa HiFi HotStart Ready Mix (Kapa Biosystems)
33. Amplify with the following PCR protocol
    a. 45 sec at 98˚ C
    b. 5 cycles of:
       15 sec at 98˚ C
       30 sec at 63˚ C

30 sec at 72˚ C

c. 1 min at 72˚ C

d. Hold at 4˚ C

34. Add 30 µl AMPure XP beads and 30 µl 30% $PEG_{8000}$ 1.25 M NaCl
35. Incubate at room temperature for 15 min.
36. Place on magnetic rack for 5 min.
37. Remove and discard supernatant. When removing supernatant do so very slowly with Pipetman being careful not to take any beads.
38. Keep sample in magnetic rack and add 200 µl of freshly prepared 80% ethanol.
39. Incubate for 30 seconds. Remove and discard all supernatant.
40. Repeat steps 38 and 39 one more time.
41. Let the beads dry at room temperature for 2 min.
42. Add 11.5 µl TE/10 and pipet up and down 10 times.
43. Incubate at room temperature for 2 min.
44. Place in magnetic rack for 5 min.
45. Transfer 10 µl of the supernatant to a new 1.5 ml tube. Again it is important to remove the supernatant very slowly being careful not to take any beads. If you accidentally remove some beads, pipet sample back into same tube and wait for the beads to separate again.
46. Prepare a 2.5% MetaPhor/SeaKem LE (3:1 ratio) agarose 1X TAE gel with ethidium bromide using small tooth comb.
47. Load 200 ng of 100 bp ladder next to each sample leaving one lane empty between sample and ladder.
48. Add 4 µl 5X loading dye (containing xylene cyanol and bromophenol blue) to sample.
49. Load sample(s) on gel with one lane between different samples and markers to avoid cross contamination.
50. Run gel at 120 V for 30 min.
51. Cut out slice of gel with clean scalpel that contains material between 250 and 450 bp and place in 1.5 ml tube.

Note: The double-stranded adapters add 121 bp to the DNA fragments. Calculate the size of your DNA fragments by running a portion of the reverse cross-linked input sample on a 2.2% agarose gel. Add 121 bp to that number and cut a gel slice containing your adapter ligated DNA. Also be sure that your gel slice is well above 121 bp, as that is where the self-ligated adapter dimers run. Cut the minimal area possible to keep your gel slice under 200 mg. A good range for most ChIP experiments is 200 bp to 550 bp.

52. Determine volume of gel slice by zeroing scale with empty tube then weighing the tube with your gel slice (1 mg = 1 µl).
53. Add 5 volumes of Qiagen Buffer QG (e.g. to a 0.2 g gel slice, add 1000 µl of QG)
54. Incubate at room temperature until gel slice has completely dissolved. Mix continuously by hand or in tube rotator.

55. Add 1 volume isopropanol.
56. Mix with Pipetman.
57. Apply 650 µl to Qiagen minElute column.
58. Spin for 30 seconds in microfuge.
59. Remove liquid from collection and reapply to minElute column a second time as described in steps 57 and 58.
60. Repeat steps 57 to 59 until you have passed entire sample through column.
61. Apply 500 µl Qiagen Buffer QG to column.
62. Spin in microfuge for 1 min.
63. Remove liquid from collection tube.
64. Apply 750 µl of Qiagen buffer PE to column
65. Let stand for 2 min
66. Spin in microfuge for 1 min
67. Remove liquid from collection tube
68. Spin in microfuge for 1 min
69. Transfer to new 1.5 ml tube
70. Add 15 µl Qiagen buffer EB
71. Let stand for 5 min.
72. Spin in microfuge for 1 min.
73. Add an additional 10 µl Qiagen buffer EB, let stand for 5 min and spin for 1 min in microfuge (both elutions are collected into the same tube)
74. Transfer eluted DNA to 0.2 ml PCR tube
75. On ice mix 24 µl Size-selected DNA (from Step 74)
    1 µl PCR primer mix (25 µM)
    25 µl 2X Kapa HiFi HotStart Ready Mix
76. Amplify with the following PCR protocol
    a. 45 sec at 98˚ C
    b. 5 to 13 cycles of:
        15 sec at 98˚ C
        30 sec at 63˚ C
        30 sec at 72˚ C
    c. 1 min at 72˚ C
    d. Hold at 4˚ C

    Note: Determine the correct number of cycles to use by following the ‘cycle quantitation protocol’ below.

77. Add 60 µl of well-mixed AMPure XP beads directly to 0.2 ml PCR tube and vortex.
78. Incubate at room temperature for 15 min.
79. Place on magnetic rack for 5 min.
80. Remove and discard supernatant. When removing supernatant do so very slowly with Pipetman being careful not to take any beads.
81. Keep sample in magnetic rack and add 200 µl of freshly prepared 80% ethanol.
82. Incubate for 30 seconds. Remove and discard all supernatant.
83. Repeat steps 81 and 82 one more time.
84. Let the beads dry at room temperature for 2 min.
85. Add 12 µl TE/10 and pipet up and down 10 times.

86. Incubate at room temperature for 2 min.
87. Place in magnetic rack for 5 min.
88. Transfer 10 µl of the supernatant to a new 1.5 ml tube.

    Attention: It is important to remove the supernatant very slowly being careful not to take any beads. If you accidentally remove some beads, pipet sample back into same tube and wait for the beads to separate again.

89. Analyze library by running 1 µl on Bioanalyzer according to manufacturer's instructions.
90. Quantitate library using library quantitation protocol.
91. Pool libraries in an equimolar ratio.

**Cycle Quantitation Protocol**

1. Mix: 8 µl $H_2O$
   1 µl of library to be amplified
   1 µl TruSeq PCR primer cocktail (25 µM)
   10 µl KAPA SYBER FAST 2x PCR master mix

2. Amplify with the following qPCR protocol
   a. 3 min at 95˚ C
   b. 20 cycles of:
      30 sec at 95˚ C
      30 sec at 63˚ C
      30 sec at 72˚ C
      plate read
3. Determine the number of cycles it took to get to 50% of amplification.

Attention: To normalize the DNA quantities so that all the samples of an experiment will amplify to an equal extent after the same number of cycles, use less DNA from the samples that amplified earlier. To calculate the number of microliter to use in the PCR reaction, use the following formula:

$$\frac{\mu l_1}{1.8^{Ct_1 - Ct_2}} = \mu l_2$$

$\mu l_1$ = number of total microliters of least concentrated sample.
$Ct_1$ = number of cycles it took least concentrated sample to reach 50% amplification.
$Ct_2$= number of cycles it took for sample being calculated to reach 50% amplification.
$\mu l_2$ = number of microliters to use in PCR reaction of sample being
calculated.

Note: If you have to do more than 18 cycles total (including pre-size selection cycles) to reach 50% amplification it is recommended that you do not proceed with sequencing.

**Library Quantitation Protocol**

1. Make the following serial dilutions in TE/10 of a library of a know concentration: 200 pM, 20 pM, 2 pM, 0.2 pM.

   Note: If you do not have a library of a known concentration, standards can be purchased from Kapa Biosystems.

2. Make three independent dilutions of your libraries at 1:1,000 and 1:10,000 in library dilution buffer.
3. Set up the following reactions using all prepared dilutions above in steps 1 and 2.
   - 4 µl diluted standards or diluted library
   - 1 µl PCR primer mix (25 µM)
   - 5 µl $H_2O$
   - 10 µl KAPA SYBER FAST 2x master mix
4. In plate set up window of the qPCR software designate the standards as 'standards' and enter their concentrations as 200, 20, 2 and 0.2. Mark the samples with libraries to be quantitated as 'samples'.
5. Amplify with the following perimeters:
   - a. 3 min at 95˚ C
   - b. 20 cycles of:
     - 30 sec at 95˚ C
     - 30 sec at 63˚ C
     - 30 sec at 72˚ C
     - plate read
6. The qPCR software calculates the efficiency of amplification of the standards, which should be between 95% and 105% and outputs the concentration of the diluted unnormalized libraries.
7. Calculate the actual concentration of the libraries using the following equation:

$$concentration\ from\ qPCR\ software \times \frac{average\ size\ of\ your\ standards\ library}{average\ size\ of\ library\ you\ are\ quantitating} \times dilution\ factor\ = actual\ library\ concentration$$

Note: a)'Concentration from your qPCR software' is the starting quantity as stated by the qPCR software, e.g. 7.3 pM.

b) 'Average size of your standards library' is the average size of the library as determined by running it on the Bioanalyzer, e.g. 275 bp.

c) 'Average size of the library you are quantitating' is the average size of the library as determined by running it on the Bioanalyzer, e.g. 275 bp.

d) 'Dilution Factor' is the amount the library was diluted, e.g. 1,000 or 10,000.

Note: It is important that the average library sizes are determined from a non-over-amplified library, as over-amplified libraries do not run true to their size on the Bioanalyzer.

**Adapter Annealing Protocol**

1. Resuspend adapter oligonucleotides at a concentration of 100 µM in:
   10 mM Tris-HCl, pH 7.8
   0.1 mM EDTA, pH 8.0
   50 mM NaCl
2. Mix 25 µl of the universal adapter with 25 µl of the indexed adapter.
3. Anneal oligos in a thermal cycler with the following program:
   a. 2 min at 95˚ C
   b. 140 cycles of
      30 sec at 95˚C (decrease temp 0.5˚ C every cycle)
   c. Hold at 4˚ C
4. Dilute annealed adaptors 1:200 in TE/10

**PCR Primer Mix**

1. Resuspend PCR Primer 1 and PCR Primer 2 in TE/10 (10 mM Tris-HCl, pH 8.0, 0.1 mM EDTA) to a concentration of 100 µM.
2. In new tube mix: 25 µl PCR Primer 1 (100 µM)
   25 µl PCR Primer 2 (100 µM)
   50 µl $H_2O$

**qPCR validation of ChIP-seq peaks**

Chromatin immunoprecipitations were performed with normal rabbit serum and anti-macroH2A1.2 antibody with the protocol described above. Each experiment was repeated three independent times. Immunoprecipitated DNA was quantitated with qPCR by comparing the threshold cycle (Ct) of the immunoprecipitated DNA to a standard curve of input DNA diluted 1:10, 1:100 and 1:1,000. qPCR reactions were set up using 4 µl immunoprecipitated DNA or the diluted input DNA standard curve, 0.5 µl 5 µM forward oligonucleotide, 0.5 µl 5 µM reverse oligonucleotide, 5 µl $H_2O$ and 10 µl KAPA SYBER Fast polymerase (Kapa Biosystems). After an initial denaturation step of 95˚C for 3 minutes, DNA was amplified with 40 cycles of 95˚C for 30 seconds, 65-72˚C for 15 seconds and 72˚C for 15 seconds. Data analysis was performed with the CFX Manager software from Bio-Rad.

**Antibody production**

Antiserum directed against amino acids 195-231 of macroH2A1.2 was produced and affinity purified as previously described[12].

**RECIPES**

<u>TE/10</u>
10 mM Tris-HCl, pH 8.0

0.1 mM EDTA

End Repair Enzyme Mix
30 µl 3 U/µl T4 DNA polymerase (NEB)
6 µl 1 U/µl Klenow Fragment (NEB)
30 µl 10 U/µl T4 DNA Polynucleotide Kinase (NEB)

Library Dilution Buffer
10 mM Tris, pH 8.0
0.05% Tween-20

**Oligonucleotide Sequences**

barcode sequence is highlighted in yellow
* = phosphorothioate bond

```
Universal
AATGATACGGCGACCACCGAGATCTACACTCTTTCCCTACACGACGCTCTTCCGATC*T

Adapter, Index 1
/5Phos/GATCGGAAGAGCACACGTCTGAACTCCAGTCACATCACGATCTCGTATGCCGTCTTCTGCTT*G

Adapter, Index 2
/5Phos/GATCGGAAGAGCACACGTCTGAACTCCAGTCACCGATGTATCTCGTATGCCGTCTTCTGCTT*G

Adapter, Index 3
/5Phos/GATCGGAAGAGCACACGTCTGAACTCCAGTCACTTAGGCATCTCGTATGCCGTCTTCTGCTT*G

Adapter, Index 4
/5Phos/GATCGGAAGAGCACACGTCTGAACTCCAGTCACTGACCAATCTCGTATGCCGTCTTCTGCTT*G

Adapter, Index 5
/5Phos/GATCGGAAGAGCACACGTCTGAACTCCAGTCACACAGTGATCTCGTATGCCGTCTTCTGCTT*G

Adapter, Index 6
/5Phos/GATCGGAAGAGCACACGTCTGAACTCCAGTCACGCCAATATCTCGTATGCCGTCTTCTGCTT*G

Adapter, Index 7
/5Phos/GATCGGAAGAGCACACGTCTGAACTCCAGTCACCAGATCATCTCGTATGCCGTCTTCTGCTT*G

Adapter, Index 8
/5Phos/GATCGGAAGAGCACACGTCTGAACTCCAGTCACACTTGAATCTCGTATGCCGTCTTCTGCTT*G

Adapter, Index 9
/5Phos/GATCGGAAGAGCACACGTCTGAACTCCAGTCACGATCAGATCTCGTATGCCGTCTTCTGCTT*G

Adapter, Index 10
/5Phos/GATCGGAAGAGCACACGTCTGAACTCCAGTCACTAGCTTATCTCGTATGCCGTCTTCTGCTT*G

Adapter, Index 11
/5Phos/GATCGGAAGAGCACACGTCTGAACTCCAGTCACGGCTACATCTCGTATGCCGTCTTCTGCTT*G

Adapter, Index 12
/5Phos/GATCGGAAGAGCACACGTCTGAACTCCAGTCACCTTGTAATCTCGTATGCCGTCTTCTGCTT*G

Adapter, Index 13
/5Phos/GATCGGAAGAGCACACGTCTGAACTCCAGTCACAGTCAAATCTCGTATGCCGTCTTCTGCTT*G

Adapter, Index 14
/5Phos/GATCGGAAGAGCACACGTCTGAACTCCAGTCACAGTTCCATCTCGTATGCCGTCTTCTGCTT*G

Adapter, Index 15
/5Phos/GATCGGAAGAGCACACGTCTGAACTCCAGTCACATGTCAATCTCGTATGCCGTCTTCTGCTT*G
```

Adapter, Index 16
/5Phos/GATCGGAAGAGCACACGTCTGAACTCCAGTCACCCGTCCATCTCGTATGCCGTCTTCTGCTT*G

Adapter, Index 18
/5Phos/GATCGGAAGAGCACACGTCTGAACTCCAGTCACGTCCGCATCTCGTATGCCGTCTTCTGCTT*G

Adapter, Index 19
/5Phos/GATCGGAAGAGCACACGTCTGAACTCCAGTCACGTGAAAATCTCGTATGCCGTCTTCTGCTT*G

PCR 1
AATGATACGGCGACCACCGA*G

PCR 2
CAAGCAGAAGACGGCATACGA*G

**Address correspondence to:** Dimitris Thanos, Institute of Molecular Biology, Genetics and Biotechnology, Biomedical Research Foundation, Academy of Athens, 4 Soranou Efesiou Street, Athens 11527, Greece. e-mail: thanos@bioacademy.gr
